# Phenotypic and Genotypic Characterization of Daptomycin-Resistant Methicillin-Resistant *Staphylococcus aureus* Strains: Relative Roles of *mprF* and *dlt* Operons

**DOI:** 10.1371/journal.pone.0107426

**Published:** 2014-09-16

**Authors:** Nagendra N. Mishra, Arnold S. Bayer, Christopher Weidenmaier, Timo Grau, Stefanie Wanner, Stefania Stefani, Viviana Cafiso, Taschia Bertuccio, Michael R. Yeaman, Cynthia C. Nast, Soo-Jin Yang

**Affiliations:** 1 Division of Infectious Diseases, Los Angeles Biomedical Research Institute at Harbor-UCLA Medical Center, Torrance, California, United States of America; 2 The David Geffen School of Medicine at UCLA, Los Angeles, California, United States of America; 3 Interfaculty Institute of Microbiology and Infection Medicine, University of Tübingen, Tübingen, Germany; 4 German Center for Infection Research (DZIF), Tübingen, Germany; 5 Department of Biomedical Sciences-Microbiology, University of Catania, Catania, Italy; 6 Division of Molecular Medicine, Harbor-UCLA Medical Center, Torrance, California, United States of America; 7 Cedars-Sinai Medical Center, Los Angeles, California, United States of America; Columbia University, College of Physicians and Surgeons, United States of America

## Abstract

Development of *in vivo* daptomycin resistance (DAP-R) among *Staphylococcus aureus* clinical isolates, in association with clinical treatment failures, has become a major therapeutic problem. This issue is especially relevant to methicillin-resistant *S. aureus* (MRSA) strains in the context of invasive endovascular infections. In the current study, we used three well-characterized and clinically-derived DAP-susceptible (DAP-S) vs. resistant (DAP-R) MRSA strain-pairs to elucidate potential genotypic mechanisms of the DAP-R phenotype. In comparison to the DAP-S parental strains, DAP-R isolates demonstrated (**i**) altered expression of two key determinants of net positive surface charge, either during exponential or stationary growth phases (i.e., dysregulation of *dltA and mprF*), (**ii**) a significant increase in the D-alanylated wall teichoic acid (WTA) content in DAP-R strains, reflecting DltA gain-in-function; (**iii**) heightened elaboration of lysinylated-phosphatidylglyderol (L-PG) in DAP-R strains, reflecting MprF gain-in-function; (**iv**) increased cell membrane (CM) fluidity, and (**v**) significantly reduced susceptibility to prototypic cationic host defense peptides of platelet and leukocyte origins. In the tested DAP-R strains, genes conferring positive surface charge were dysregulated, and their functionality altered. However, there were no correlations between relative surface positive charge or cell wall thickness and the observed DAP-R phenotype. Thus, charge repulsion mechanisms via altered surface charge may not be sufficient to explain the DAP-R outcome. Instead, changes in the compositional or biophysical order of the DAP CM target of such DAP-R strains (i.e., increased fluidity) may be essential to this phenotype. Taken together, DAP-R in *S. aureus* appears to involve multi-factorial and strain-specific adaptive mechanisms.

## Introduction

Daptomycin (**DAP**) is a calcium-dependent lipopeptide antibiotic with potent bactericidal effects against most Gram-positive pathogens [1], [2]. Since its release for use in 2003 for skin and soft tissue infections, followed by approval for *Staphylococcus aureus* bacteremia and right-sided endocarditis in 2006, DAP has become a key antibiotic for invasive staphylococcal infections. This is especially relevant to methicillin-resistant *S. aureus* (**MRSA**) infections in the era of rising vancomycin MICs associated with clinical treatment failures [3]–[5], as well as vancomycin (VAN)-intermediate *S. aureus* (VISA)-related infections [2], [5], [6]. Recently, there have been an alarming number of reports of both *S. aureus* and enterococcal DAP-resistant (**DAP-R**) strains emerging during DAP treatment failures [7]–[12]. The mechanisms by which such organisms develop resistance to the microbicidal effects of DAP are likely multi-modal and organism-dependent [9], [13]–[18]. Thus, integrative genotypic and phenotypic profiles among DAP-R *S. aureus* strains differ substantially from those defined among DAP-R enterococci [9], [10], [13], [14], [16]–[18]. Moreover, although a principle mechanism of DAP action appears to be perturbation of the bacterial cell membrane (**CM**), key impacts upon cell wall (**CW**) turnover processes seem to play a cardinal role in this regard [19]–[21]. Since DAP unambiguously requires calcium complexing to execute its CM-targeting and subsequent bactericidal effects, this agent acts similarly to cationic antimicrobial peptides involved in innate host defenses (e.g., from PMNs and platelets [22], [23]). The range of potential phenotypic adaptations that may be associated with staphylococcal DAP-R include: **i**) increased positive surface charge (‘charge-repulsion hypothesis’) [9], [17], [18], [24]; **ii**) altered CM fatty acid composition resulting in altered CM fluidity [14], [25], [26] (‘membrane order hypothesis’); **iii**) increased CM carotenoid pigment content yielding very rigid CMs [27]; **iv**) a combination of factors; for example, enhanced CM content of positively-charged phospholipids, as well as increased D-alanylation of CW teichoic acid, resulting in reduced affinity of DAP to the CM target [28]; and **v**) augmented synthesis of CW teichoic acid, creating a thickened CW phenotype and a putative mechanical barrier to DAP penetration to reach its CM target [28], [29].

The above phenotypic correlates of DAP-R in *S. aureus* point to specific genes as potentially involved in this resistance process. In this regard, the *mprF* operon (involved in lysinylation of CM phosphatidylglycerol [PG] to synthesize lysyl-PG [L-PG], as well as to translocate this latter positively-charged species to the outer CM) and the *dlt* operon (responsible for the D-alanylation of CW teichoic acid) have received particular attention [17], [24], [28]–[33]. However, studies have varied widely in ascribing causality to these two loci in DAP-R *S. aureus* strains, since a broad range of genotypic correlates with the DAP-R phenotype have emerged, including: **i**) acquisition of gain-in-function single nucleotide polymorphisms (**SNPs**) in the *mprF* ORF [9], [11], [24], [25], [34]; **ii**) over-expression of *dlt* genes [17]; and **iii**) dysregulation of both *mprF* and *dlt* expression profiles [17], [24], [29]. Recently, Cafiso et al [31] studied three clinical MRSA strain-sets in which DAP-R evolved during treatment, and were able to pinpoint *dltA* overexpression (especially in strains acquiring a concomitant SNP in *mprF*) as potentially causal in the DAP-R phenotype. This intriguing investigation was, however, somewhat limited, in that within the DAP-S and DAP-R strain-sets: **i**) there was no growth phase-dependent gene expression profiling performed; **ii**) there were no phenotypic correlates of *dlt* and *mprF* over-expression studied, especially surface charge measurements; **iii**) specific phenotypic readouts of *dlt* and *mprF* over-expression were not quantified (i.e., CW D-alanylation and CM L-PG synthesis and/or translocation, respectively [25], [28], [32]); **iv**) other parameters of CM physiology previously associated with DAP-R (e.g., CM fluidity and fatty acid contents) were not queried [9], [27], [35]; and **v**) assessment of DAP-R isolates for *in vitro* ‘cross-resistance’ with several prototypical host defense cationic peptides (**HDPs**) involved in staphylococcal pathogenesis was not performed [9], [18], [25], [35]. The latter cross-resistance phenomenon has been well-chronicled for other DAP-R *S. aureus* strains [25]. The current study, employing the same previously published DAP-S/DAP-R strain-pairs [31], was designed to address these limitations, and to further characterize potential mechanisms of DAP-R in these strains.

## Methods and Materials

### Bacterial strains

The three clinically-derived DAP-S/DAP-R MRSA strain-pairs used in the current study (**Table 1**) have been described in detail before [31]. Briefly, the epidemiologically unrelated stran-pairs were isolated from different patients, were from different clinical infection types (skin and soft tissue; bacteremia), and were from three different Italian hospitals. All three patients had received and failed therapy with the glycopeptides antibiotic, teicoplanin, and were subsequently treated with DAP [31]. Each strain-pair included an initial pre-DAP therapy DAP-S MRSA strain and an isogenic DAP-R isolate obtained during DAP therapy. Isogenicity of each strain-pair was confirmed by identical outcomes of several discriminative genotypic assays within each DAP-S and DAP-R strain-pair, including: pulse field gel electrophoresis typing (PFGE), *agr* typing, multi-locus sequence typing and staphylococcal casette chromosome (SCC*mec*) typing. The three strain-pairs were previously characterized for mutations in the *mprF* genes. Two of the three DAP-R variants within each strain-pair exhibited a non-synonomous SNP in the *mprF* ORF (**Table 1**), while the third strain-set did not. Of note, these two SNPs occurred in previously described “hotspots” within the *mprF* locus associated with MprF gains-in-function and the DAP-R phenotype [9], [32], [34]. The above genotypic data for these three strain-pairs have been previously published [31].

**Table 1:** Bacterial strains examined in the current studies. *

Strain	ST type	SCC*mec*	PFGE	*agr* type	DAP MICs	VANC MICs	*mprF* SNPs
**1A**	**398**	**Iva**	**Apal/α1**	**I**	**≤0.25**	**1**	**-**
**1C**	**398**	**Iva**	**Apal/α1**	**I**	**4**	**2**	**S295L**
**2B**	**5**	**II**	**USA100**	**II**	**0.5**	**1**	**-**
**2C**	**5**	**II**	**USA100**	**II**	**2**	**2**	**T345I**
**3A**	**22**	**IV**	**G1**	**I**	**0.5**	**1**	**-**
**3B**	**22**	**IV**	**G1**	**I**	**4**	**2**	**-**

*These data have been previously published [31]; DAP  =  daptomycin; VAN  =  vancomycin; SNPs  =  single nucleotide polymorphisms.

### MIC testing

Oxacillin, vancomycin and DAP MICs were performed according to CLSI guidelines [36]. These data have been previously reported (**Table 1**) [31].

### Surface charge

The cytochrome *c* binding assay was performed as a surrogate measure of the relative net positive surface charge of the strain-pairs as described previously [17], [18], [37]. Briefly, cells were grown overnight in TSB media, washed with 20 mM MOPS buffer (pH 7.0) three times and resuspended in the same buffer at OD_578_ = 1.0. Cells were incubated with 0.5 mg/ml cytochrome *c* for 10 minutes and the amount of cytochrome *c* remaining in the supernatant was determined spectrophotometrically at OD_530_ nm. The more unbound cytochrome *c* that was detected in the supernatant, the more net positively charged the bacterial surface. Data were converted and expressed as mean (± SD) amount of bound cytochrome *c*. At least three independent runs were performed on separate days.

### Wall teichoic acid (WTA) isolation and purification

*S. aureus* CW and WTA were specifically isolated as described in detail before [39], [40]. In brief, bacteria were grown overnight in B-Medium (1% peptone, 0.5% yeast extract, 0.1% glucose, 0.5% NaCl and 0.1% K_2_HPO_4_) containing 0.25% (wt/vol) glucose, washed twice in sodium acetate buffer (20 mM, pH 4.7) and disrupted in the same buffer with glass beads for 1 h on ice in a cell disruptor (Euler). Protein-free CW was isolated, and WTA was released by treatment with 5% trichloroacetic acid in sodium acetate buffer for 4 h at 60°C. The CWs were removed by centrifugation and WTA was quantified by determining its inorganic phosphate (Pi) content as described before [40]. The isolation was performed in triplicate for each strain, and assayed in triplicate for their respective Pi content.

### Quantification of D-alanylated WTA content

The D-alanylation of the WTA polymers was assayed and quantified as described before [41]. In brief, D-alanine esters were hydrolyzed by a mild alkaline hydrolysis carried out at 37°C for 1 h in 0.1 M NaOH. The supernatant was neutralized, dried under vacuum, and used for pre-column derivatization with Marfey's reagent (1-fluoro-2, 4-dinitrophenyl-5-L-alanine amide; Sigma). Amino acid derivates (detection at 340 nm) were then separated as described before and analyzed with the ChemStation software. Data were expressed as percent of WTA (± SD) that was D-alanylated. A minimum of three independent runs was performed.

### Cell wall (CW) thickness

The CW thickness of study strains were measured by transmission electron microscopy (TEM; [25], [26]). The mean CW thickness (nm ± SD) of 100 cells was determined for the strains at a constant magnification of 190,000× (JEOL, Model# 100CX, Tokyo, Japan) using digital image capture and morphometric measurement (Advanced Microscopy Techniques v54, Danvers, MA).

### Host defense peptides (HDPs)

Thrombin-induced platelet microbicidal proteins (tPMPs) were obtained from thrombin-stimulated rabbit platelets as previously described [43]. This preparation contains several tPMPs, but predominantly tPMP-1. The bioequivalency (activity in µg/ml) of the tPMP preparation was determined as detailed before, using a *Bacillus subtilis* bioassay [9]. Purified human neutrophil defensin-1 (hNP-1) and LL-37 (prevalent in neutrophils and skin epithelium [22], [44], [45]) were purchased from Peptides International (Louisville, KY). RP-1 (a synthetic congener of the microbicidal domain of the platelet factor-4 family of kinocidins) was synthesized as previously detailed [46].

### Susceptibilities to HDPs

For tPMPs and RP-1, a microtiter bactericidal assay was carried out in minimal liquid nutrient medium (Eagles minimal essential media [MEM]) in appropriate buffers [25]; the hNP-1 and LL-37 killing assays were performed in 1% BHI +10 mM potassium phosphate buffer (PPB). A final bacterial inoculum of 10^3^ stationary phase CFU was employed. The peptide concentrations used in the 2 h killing assays were: 1.5 or 2.0 µg/ml bioactivity equivalent for tPMPs; 5 or 10 for hNP-1; and 0.5 and 1 µg/ml for RP-1 and LL-37. After extensive pilot studies, these peptide concentrations were selected based on: (**i**) sub-lethality, with <50% reductions in counts of the parental DAP-susceptible (DAP-S) strain; and (**ii**) encompassing peptide concentrations used in prior investigations of HDP:*S. aureus* interactions [25]. After 2 h peptide exposure, samples were obtained and processed for quantitative culture to evaluate the extent of killing by each HDP condition. Final data were expressed as mean (± SD) percent survival rate. Since there is no *bona fide* “resistance” breakpoint for HDPs, the mean percent survival (± SD) was statistically evaluated for potential correlates of HDP and DAP susceptibility profiles. Data included a minimum of three experiments performed on separate days.

### CM phospholipid (PL) and amino-PL translocation (asymmetry)

To investigate potential correlates between *mprF* polymorphisms and CM features, PLs were extracted from study strains under specific test conditions as described [26]. The major CM PLs of *S. aureus* (PG; L-PG and cardiolipin [CL]) were separated by two-dimensional thin-layer chromatography (2-D TLC) using Silica 60 F254 HPTLC plates (Merck). Fluorescamine labeling (a fluorophore which binds only to positively charged PLs, such as L-PG, and which does not penetrate the outer CM leaflet), combined with ninhydrin staining localization, was used within the 2-D TLC plate assay to assess the translocation of L-PG between the inner-to-outer CM bilayer [13], [17], [26]. First-dimension chloroform-methanol–25% ammonium hydroxide (65∶25∶6, by volume) in the vertical orientation and second-dimension chloroform:water:methanol:glacial acetic acid:acetone (45∶4∶8∶9∶16, by volume) in the horizontal orientation were used for the separation of the PLs for further quantitation by phosphate estimation. PL identity was confirmed using known PL standards. For quantitative analysis, resulting isolated PLs (identified by iodine staining) were digested at 180°C for 3 h with 0.3 ml 70% perchloric acid and the oxidized derivatives quantified spectrophotometrically at OD_660_.

### CM fatty acid composition

Given the impact of fatty acid composition on CM adaptability to stress, the comparative fatty acid profiles of the DAP-S/DAP-R strain-pairs were determined. Approximately 20 mg of bacterial cells were harvested from late log phase growth preparations, and then saponified, methylated, and fatty acid esters extracted into hexane as described previously [17], [26]. The resulting methyl ester mixtures were separated by an Agilent 5890 dual-tower gas chromatograph. Fatty acids were identified and quantified by a microbial identification system (Sherlock 4.5; courtesy of Microbial ID Inc., Newark, DE) [17], [26].

### CM fluidity

CM fluidity was determined by fluorescence polarization spectrofluorometry as detailed previously [9], [25], [26] using the fluorescent probe 1,6-diphenyl-1,3,5-hexatriene (DPH). An inverse relationship exists between polarization indices and the degree of CM order (i.e., lower polarization indices [PI value] denotes a greater CM fluidity) [9], [25], [26]. To address day-to-day biological variability inherent to CM dynamics, these assays were performed a minimum of six times for each strain on separate days.

### DNA isolation and targeted *mprF, dltA, and dltB* sequencing

Genomic DNA was isolated from *S. aureus* using the method of Dyer and Iandolo [47]. PCR amplification of the *mprF* ORF was performed as we have previously described, using the primers, *mprF*-F-bam (5′-CCCGGATCCAATTAGAATTGATGTGAAAAAATG-3′) and *mprF*-R-sph (5′-CCCGCATGCAGCGCTTCAGGCATAACTGT-3′) [24].

PCR amplification of the *dltA* and *dltB* ORFs were performed as we have described previously with primer pairs dltA-ORF-F (5′-CAGTGGCGACACACACAATA-3′) and dltA-ORF-R (5′-GACTGGTAATAATGCAATTAAAGCAA-3′), dltB-ORF-F (5′-TGGAACAATTGCCATTGACTT-3′) and dltB-ORF-R (5′-TCCAACTGTTTGGAAAGA ATCA-3′), respectively [17]. DNA sequencing of the *mprF and dlt genes* was kindly performed at City of Hope, Duarte, CA.

### RNA isolation and qRT-PCR analysis for *mprF* and *dltA* transcription

For RNA isolation, fresh overnight cultures of *S. aureus* strains were used to inoculate NZY broth to an optical density at 600 nm (OD_600_) of 0.1. Cells were harvested during both exponential growth (2.5 h; OD = 0.5) and stationary phase (12 h). Total RNA was isolated from the cell pellets by using the RNeasy kit (Qiagen, Valencia, CA) and the FASTPREP FP120 instrument (BIO 101, Vista, CA), according to the manufacturer's recommended protocols.

Quantitative real time PCR assay was carried out as detailed previously [29], [48]. Briefly, 1 µg of DNase-treated RNA was reverse transcribed using the SuperScript III first-strand synthesis kit (Invitrogen) according to the manufacturer's protocols. Quantification of cDNA levels was performed following the instructions of the Power SYBR green master mix kit (Applied Biosystems) on an ABI PRISM 7000 sequence detection system (Applied Biosystems) or on a LightCycler using the Quanti Fast SYBR green real-time (RT)-PCR kit (Qiagen). The *mprF, dltA,* and *gyrB* genes were detected using specific primers and computational methods as described before [29], [48].

## Results

### HDP susceptibility profiles

As anticipated, the peptides exerted distinct efficacies against the study strains (**Table 2**). RP-1 exhibited the greatest anti-staphylococcal activity, including against stain 2C which exhibited high level resistance to other peptides (**Table 2**). There was a general trend for all HDPs tested, showing that the DAP-R isolate in each strain-pair was significantly more resistant to peptide-mediated killing than its DAP-S parental strain. This difference in HDP killing profiles between DAP-S/DAP-R strain-pairs was most dramatic for tPMPs and RP-1 of platelet origin, and LL-37 from leukocytes, while being the least notable for hNP-1 (by far the least active of the peptides against these study strains).

**Table 2:** *In vitro* susceptibility to killing by cationic host defense peptides and the congener, RP-1.

Strain	% survival (mean ± SD) after 2-h exposure to:							
	tPMPs (2 µg/ml)	tPMPs (1 µg/ml)	LL-37 (1 µg/ml)	LL-37 (0.5 µg/ml)	hNP-1 (20 µg/ml)	hNP-1 (10 µg/ml)	RP-1 (1 µg/ml)	RP-1 (0.5 µg/ml)
**1A**	**2.5±2.0**	**4.7±3.8**	**0.5±0.9**	**11.7±6.0**	**46.3±18.9**	**72.6±22.2**	**4.3±3.2**	**15.9±9.6**
**1C**	**18.0±13.5** **	**38.1±15.3** *	**36.1±17.6** **	**59.0±19.0** **	**68.2±19.2** *	**79.2±19.6**	**22.3±12.5** **	**52.7±14.7** **
**2A**	**4.1±3.2**	**19.4±9.9**	**0.5±0.7**	**13.1±11.8**	**44.20±30.4**	**60.8±26.1**	**1.4±1.2**	**11.3±5.4**
**2C**	**83.0±8.4** **	**90.8±11.4** **	**22.4±12.6** **	**65.9±26.1** **	**66.0±18.0**	**92.1±17.5** *	**4.7±5.0**	**26.1±15.1** *
**3A**	**7.4±5.8**	**25.9±11.9**	**1.8±2.5**	**62.0±20.8**	**35.6±26.1**	**60.3±24.2**	**1.0±1.5**	**17.3±8.5**
**3B**	**80.4±13.5** **	**86.7±12.1** **	**49.9±19.9** **	**99.6±10.4** **	**88.7±15.2** **	**91.1±8.6** **	**10.9±9.6** *	**34.5±12.2** *

*P<0.05;

**P<0.01 vs the DAP-S parental strains.

### Surface charge (Figure 1)

**Figure 1:**
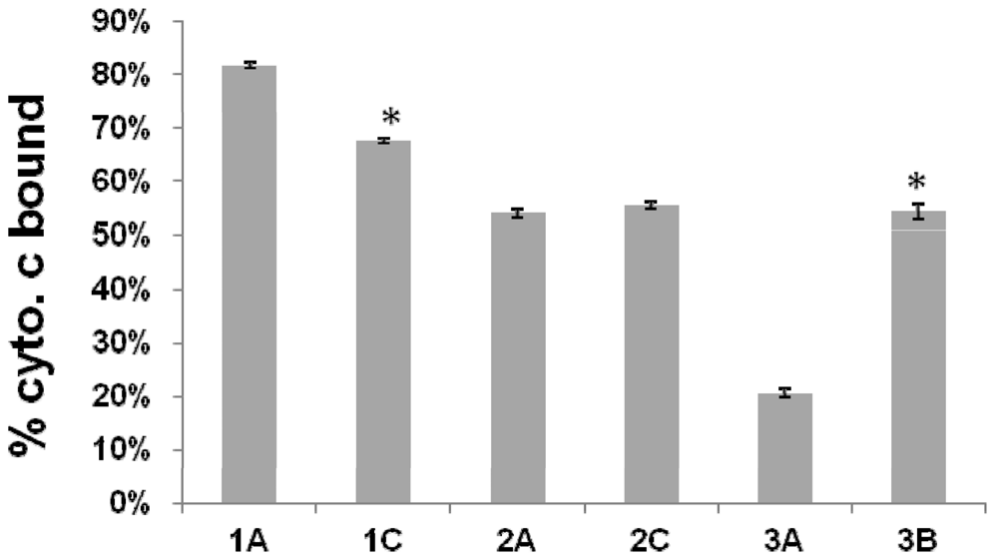
Relative positive surface charge by cytochrome *c* binding. The graph shows percent of cytochrome *c* bound after 10 min of incubation with *S. aureus* cells at room temperature. Data represent the means and standard deviations from three independent experiments. *P<0.01 vs DAP-S parental strains.

Outcomes of relative positive surface charge comparisons between DAP-S and DAP-R isolates varied substantially between strain-pairs. For example, for strain-pair 1, the DAP-R isolate (1C) exhibited a significantly more relative positive surface charge than its parental DAP-S isolate (1A). In contrast, the 3A/3B strain pair showed the exact opposite surface charge relationships; the DAP-S strain 3A bound more cytochrome C than did strain 3C. The 2A/2C strain set had virtually identical surface charge readouts.

### Wall teichoic acid (WTA) content (Figure 2)

**Figure 2:**
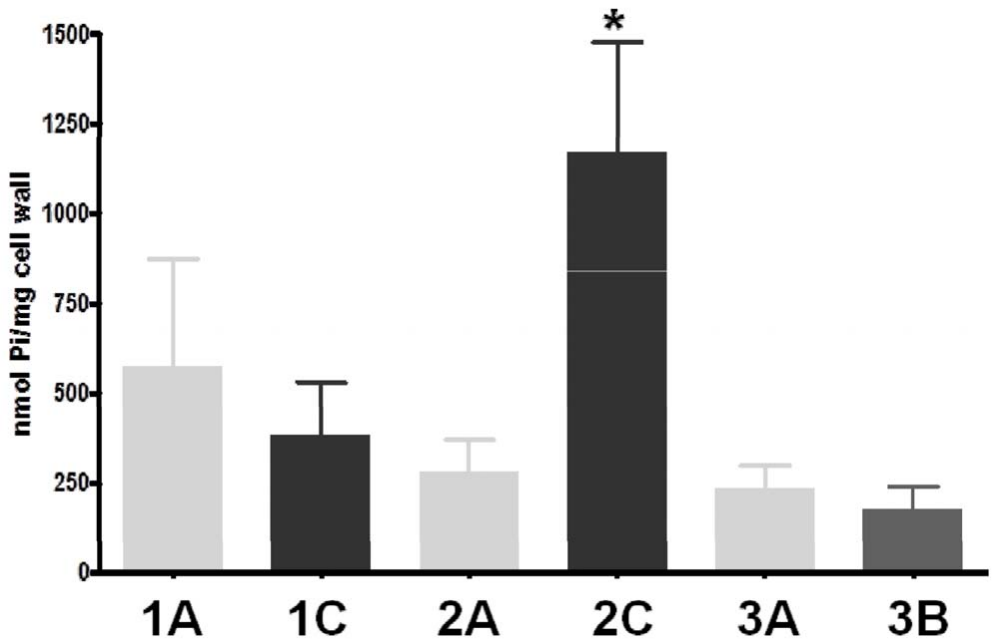
Cell wall teichoic acid (WTA) contents of the strain sets. Dry mass of cell wall was quantified as [g dry weight/g wet weight]. The amount of WTA was determined by a colorimetric assay and expressed as [nmol Pi/mg cell wall; n = 5]. Statistical analysis was performed by Student's t-test. *P<0.05 vs DAP-S parental strain.

A significant increase in the amount of WTA in the CW of the DAP-S/DAP-R strain-pair 2A/2C was detected, with the DAP-R strain producing significantly more WTA than the respective DAP-S strain (*P*<0.05). In contrast, in the strain-pairs 1A/1C and 3A/3B, no difference in WTA amount in the CWs of the respective DAP-R strains was detected.

### WTA D-alanylation (Figure 3)

**Figure 3:**
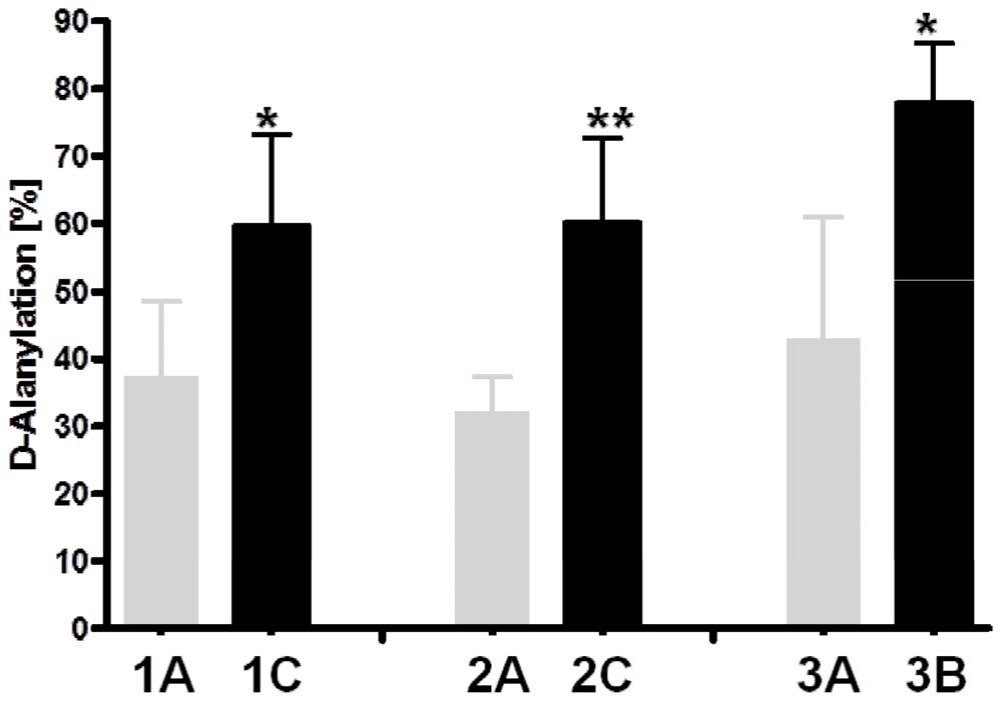
WTA D-alanylation in the DAP-S/DAP-R strain pairs. The rate of D-alanylation of WTA repeating units was determined by HPLC (n = 3). Statistical analysis was performed by Student's t-test. *P<0.05; **P<0.01 vs DAP-S parental strains.

In addition to the significant increase in overall WTA content in the DAP-R strain 2C, there was also a substantial difference in the proportion of WTA that was D-alanylated when comparing strain 2C to its isogenic DAP-S strain 2A. Interestingly, the percentage of D-alanine contained within the WTA (nmol D-alanine/nmol Pi) of the DAP-R strains that did not exhibit an increase in WTA content (1C and 3B) was also significantly higher than that observed in their respective DAP-S parental strains (1A and 3A). These latter data speak to a clear-cut gain-in-function of the DltA gene [28], [29].

### CM fatty acid profiles

Fatty acid compositional data for the three strain-pairs are shown in **Table 3** (a detailed Table enumerating each specific fatty acid species is included as **Table S1**). In two of the three strain-pairs (**1A–1C and 2A–2C**), fatty acid composition exhibited a rather similar overall profile, including proportionality of iso- and ante-iso branched chain fatty acids, unsaturated fatty acids (UFAs), and acyl chain-length profiles. Thus, the increases in CM fluidity observed in the above two DAP-R *S. aureus* strains could not be directly correlated to changes in the proportion of key fatty acids known to impact CM fluidity; i.e, iso- vs anteiso branch chain fatty acids, and saturated vs unsaturated fatty acids). In contrast, in one DAP-R strain (**3B**), fatty acid analyses demonstrated a significant increase in the proportion of anteiso-branch chain species (vs iso-branch chain species) and decrease in saturated fatty acids as compared to its respective parental DAP-S strain (**3A**) (*P*<0.05). In this strain-pair, the total proportions of unsaturated fatty acids were not altered, although an additional fatty acid species (18:2ω6, 9c) was present in DAP-R strain 3B. This increase in anteiso-branch chain species appeared to correspond with a significant reduction in the major iso-branched chain and saturated fatty acid (SFAs) species, respectively, in this DAP-R strain (*P*<0.05). These proportionality perturbations in this latter DAP-R strain provided a potential explanation for the observed increment in CM fluidity in this latter strain (see below).

**Table 3:** Comparative fatty acid (FA) compositions of *S. aureus* study strains.

Strains	FA species (% of fatty acid composition ± SD)			
	Iso-BCFA	Anteiso-BCFA	UFAs	SFAs
**1A**	**14.8±0.9**	**58.6±0.2**	**8.6±0.9**	**15.2±0.2**
**1C**	**15.9±0.9**	**57.7±0.2**	**9.3±0.9**	**14.5±0.1**
**2A**	**17.5±0.7**	**55.7±2.1**	**9.0±1.3**	**15.1±1.3**
**2C**	**16.3±0.1**	**55.2±0.1**	**9.2±0.1**	**16.1±0.1**
**3A**	**15.5±0.3**	**53.5±0.3**	**8.0±0.3**	**19.8±0.3**
**3B**	**13.5±0.3** *	**62.9±0.8** *	**7.6±0.5**	**14.2±0.6** *

BCFAs =  Branched chain fatty acids; UFAs  =  unsaturated fatty acids;

SFAs =  Saturated fatty acids.

*P<0.05 vs. respective parental strain.

### CM phospholipid (PL) content

Since *mprF* regulates the lysinylation of PG to yield L-PG, as well as its subsequent outer CM translocation, we compared these parameters in the three DAP-S/DAP-R strain pairs. As noted in **Table 4**, in strain pairs 1A/1C and 2A/2C, the DAP-R isolates contained significantly more L-PG than their respective DAP-S parental strains. This outcome was associated with a significant reduction in PG content in both DAP-R isolates. For the third strain-pair (3A/3B), an opposite readout was observed, with the DAP-R strain containing less L-PG than its DAP-S parental strain. Of interest, the outer CM flipping profiles for L-PG did not differ substantially in comparing any DAP-S strain to their respective DAP-R isolates. Thus, the net CM L-PG profile of our study strains reflected the rate of L-PG synthesis, rather than a difference in the extent of CM PL flipping.

**Table 4:** Comparative cell membrane phospholipid profiles of study strains.

Strains	% of total phospholipids ± SD				
	Inner-LPG	Outer-LPG	Total LPG	PG	CL
**1A**	**11±3.3**	**2.0±1.0**	**13±2.8**	**81±2.8**	**6±1.4**
**1C**	**22±5.5** **	**5±2.9** *	**26±7.3** **	**68±10.2** **	**6±3.1**
**2A**	**9±2.5**	**2±1.2**	**11±1.7**	**84±2.3**	**5±2.1**
**2C**	**19±4.0** **	**4±4.0**	**24±6.4** **	**67±8.9** **	**9±5.5**
**3A**	**19±3.3**	**3±0.8**	**22±3.9**	**69±5.4**	**9±1.9**
**3B**	**13±2.5** **	**3±1.3**	**16±3.2** *	**80±3.6** **	**4±1.7** **

Abbreviations: LPG, lysyl-phosphatydylglycerol; PG, phosphatidylglycerol; CL, cardiolipin.

*P<0.05;

**P<0.01 vs respective DAP-S parental strains.

### CM fluidity (Figure 4)

**Figure 4:**
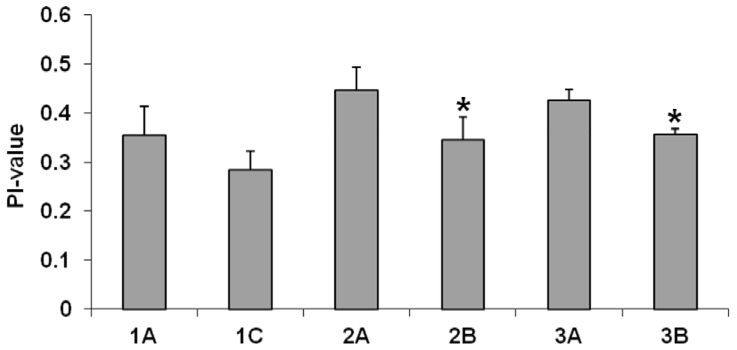
Cell membrane fluidity by fluorescence polarization indices. CM fluidity was determined by using the fluorescent prode 1,6-diphenyl-1,3,5-hexatriene (DPH). These assays were performed a minimum of six times for each strain. Note: Lower polarization indices  =  higher CM fluidity. *P<0.05 vs DAP-S parental strains.

The polarization indices (PI values) for all three DAP-R isolates were substantially lower than that of their respective DAP-S parental strains, indicating that the CMs of these DAP-R isolates were more fluid than their DAP-S counterparts. These differences reached statistical significance for strain-pairs 2A–2C and 3A–3B.

### *mprF* expression profiles

**Figure 5:**
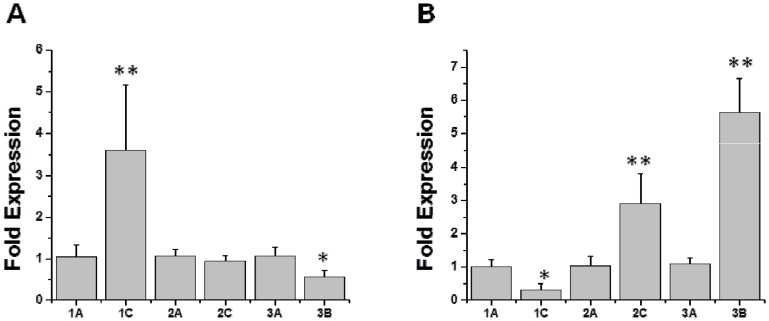
Relative transcription level of *mprF* during exponential (A) and stationary (B) growth phase. RNA samples were isolated from exponential- and stationary-phase cultures of the strains and were subjected to qRT-PCR to detect transcription of *mprF* and *gyrA*. EXPO  =  exponential growth phase; ST  =  stationary growth phase; Fold expression  =  compared to *gyrB* gene, with parental strain fold-expression set at “1”. **P<0.01 vs DAP-S parental strains.

**Figures 5A and 5B** demonstrate the relative expression profiles for *mprF* during exponential vs stationary growth phases in these strain-pairs. During exponential growth, the DAP-R strain 1C showed ∼4-fold increase in *mprF* expression vs its DAP-S parental strain. Expression profiles for the other two strain pairs did not demonstrate such increases among the DAP-R isolates. In contrast, at stationary growth (when *mprF* expression is generally minimal [24]), the two other DAP-R isolates (2C and 3B) exhibited ∼3 and ∼6-fold increases, respectively, in *mprF* expression as compared to their DAP-S parental strains. Of note, this stationary phase dysregulation of *mprF* expression in DAP-R strain, 3B, occurred despite the lack of a definable SNP in *mprF* in this isolate.

### *dltA* expression profiles

**Figure 6:**
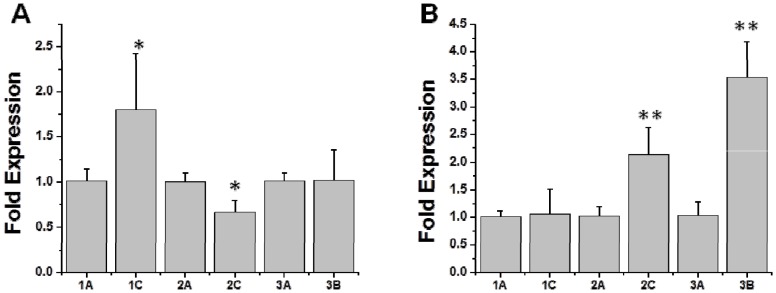
Relative transcription level of *dltA* during exponential (A) and stationary (B) growth phase. RNA samples were isolated from exponential- and stationary-phase cultures of the strains and were subjected to RT-PCR to detect transcription of *dltA* and *gyrA*. EXPO  =  exponential growth phase; ST  =  stationary growth phase; Fold expression  =  compared to *gyrB* gene, with parental strain fold-expression set at “1”. *P<0.05; **P<0.01 vs DAP-S parental strains.

As shown in **Figures 6A and 6B**, growth phase-dependent *dltA* expression profiles for the DAP-S vs DAP-R strain-pairs showed remarkably similar metrics as for the *mprF* profiles above. Thus, *dltA* expression was increased in DAP-R isolates as compared to their DAP-S parental strain at either exponential or stationary phases of growth.

### *dltA* and *dltB* sequencing

Although a few nucleotide sequence polymorphisms were observed within the *dltA* and *dltB* among the 3 pairs of the strains, only the DAP-R 3B strain revealed one non-synonymous SNP (T-to-C at 763) in the *dltA* gene as compared to its DAP-S parental strain. This mutation yields a phenylalanine-to-leucine (F255L) amino acid substitution (data not shown). Sequence analyses of *dltB* in the three pairs revealed no differences between the DAP-S and DAP-R pairs.

## Discussion

Recently, Cafiso et al [31] have provided further evidence for the potential role of both the CW, and specifically the *dlt* operon in the DAP-R phenotype. These investigators documented the exponential growth-phase enhancement of *dlt* expression in three DAP-R MRSA strains which emerged during DAP therapy, in the presence or absence of *mprF* ‘hot spot’ SNPs. These authors proposed that *dlt* over-expression is a common pathway of DAP-R. Of note, their data regarding the potential role of *dlt* overexpression and DAP-R mirrors recent findings from our own laboratories [28], [29]. However, the investigation of Cafiso et al did not elucidate potential phenotypic or genotypic mechanism(s) by which *dlt* over-expression could be causal in DAP-R.

The current study, utilizing these same DAP-S/DAP-R strain-pairs sought to extend and adjudicate the studies of Cafiso et al [31]. A number of interesting observations emerged from this investigation. *First*, as has been seen previously in selected DAP-R MSSA and MRSA strains [17], [28], [29], [51], *dltA* expression is frequently dysregulated, either by enhanced exponential phase or increased stationary phase transcription profiles. Of interest, *dltA* expression is generally quite minimal at stationary phase in DAP-S *S. aureus* strains [17]. Furthermore, despite these notable differences in *dltA* transcription, only one of the three DAP-R isolates exhibited a non-synonymous SNP in *dltA*. Phenotypically, all three DAP-R strains demonstrated a significant increase in their WTA D-alanylation content, reflecting DltA gains in funtionality. Of interest, concomitant increases in CW thickness were not observed in any of the three DAP-R strains (data not shown).

*Second*, *mprF* expression profiles were remarkably similar to those of *dltA* above in all three strain-pairs, whether or not ‘hot spot’ non-synonymous *mprF* SNPs were identified in the DAP-R isolates. The observation that *mprF* transcriptional dysregulation occurred despite the absence of such SNPs supports the notion that regulatory genes outside of *mprF* can affect its expression profile. As expected, phenotypically, in the two DAP-R strains with *mprF* SNPs, enhanced amounts of L-PG synthesis were detected as compared to their respective DAP-S parental isolates. In the remaining DAP-R strain, despite overt *mprF* dysregulation, L-PG synthesis was not increased. This finding is reminiscent of data in selected MSSA DAP-R isolates [9], [24].

*Third*, one prevailing concept that has been put forward to explain the DAP-R phenotype in *S. aureus* has been perturbations in relative cell surface positive charge, creating a “charge repulsion” scenario against calcium complexed-DAP. However, despite the increases in *dltA* transcription, D-alanylation of WTA and L-PG synthesis among the three DAP-R strains, this did not translate into consistent enhancement of surface positive charge in these isolates. These data refute charge repulsion as a principal or comprehensive explanation for the DAP-R phenotype in these strain-pairs.

*Fourth*, all three DAP-R strains demonstrated substantial increases in their CM fluidity properties as compared to their respective DAP-S parental strains. Altered CM fluidity has been closely linked to resistance to killing by cationic antimicrobial peptides in general [9], [19], [25]. Such outcomes are presumed to relate to perturbations in the ability of such peptides to interact with and/or insert within target CMs. It is hypothesized that there are biophysical and/or biochemical ‘optima’ in the CM microenvironments of *S. aureus* strains, making them more or less susceptible to cognate host defense peptides (HDPs) with specific structure-activity signatures. Thus, CMs that exhibit high degrees of rigidity or fluidity are relatively resistant to interactions with specific HDPs that likely rely most upon more intermediate degrees of CM order [25], [27]. The global impact of altered fluidity in the current DAP-R vs DAP-S strains is underscored by the *in vitro* “cross-resistance” of the DAP-R strains to cationic HDPs which are structurally distinct from DAP, including prototypical peptides from mammalian platelets and leukocytes. Such cross-resistance between DAP and HDPs has been well-chronicled in recent studies amongst both staphylococci and enterococci [16], [25], [34], [52], [53]. The mechanism(s) of enhanced fluidity in the current three DAP-R strains is not entirely clear, although in one DAP-R isolate the ratio of anteiso-branch chain fatty acids (BCFAs) was significantly increased as compared to its DAP-S parental strain. Such iso-to-anteiso-BCFA shifts have been previously associated with increased CM fluidity properties in *Bacillus subtilis* in response to cold shock [54].

Thus, in summary, the ultimate mechanism(s) of DAP-R in *S. aureus* is highly likely to be multi-factorial and strain-specific. Although over-expression and dysregulation of *dltA* transcription may well be identifiable in selected DAP-R *S. aureus* strains and correlated with increased WTA D-alanylation, its subsequent potential phenotypic consequences (i.e., changes in relative surface charge) were not sufficient to entirely explain the DAP-R phenotype in these strains.

## Supporting Information

Table S1 Distinct fatty acid (FA) compositions of strain pairs.
(DOC)
