# Supplementary material for: Phenotypic and Genotypic Characterization of Daptomycin-Resistant Methicillin-Resistant Staphylococcus aureus Strains: Relative Roles of mprF and dlt Operons
Source: PLoS One. 2014 Sep 16;9(9):e107426. doi: 10.1371/journal.pone.0107426 (PMC4166420; doi:10.1371/journal.pone.0107426)
Supplement: Table S1 — Distinct fatty acid (FA) compositions of strain pairs. (DOC) [file pone.0107426.s001.doc]

**Supplemental Table S1.** Distinct fatty acid (FA) compositions of strain pairs.

| **% of FA composition (Iso-BCFA = Iso-branch chain FAs; Anteiso-BCFAs; SFA = Saturated FAs; and UFAs = Unsaturated FAs)** | | | | | | |
| --- | --- | --- | --- | --- | --- | --- |
|  | **1A** | **1C** | **2A** | **2C** | **3A** | **3B** |
| **Iso-BCFA** |  |  |  |  |  |  |
| **14:0 iso** | **1.2±0.1** | **1.0±0.2** | **0.8±0.1** | **0.7±0.4** | **1.6±0.0** | **0.6±0.0*** |
| **15:0 iso** | **5.3±0.1** | **6.5±0.2*** | **7.0±0.1** | **7.0±2.7** | **4.4±0.1** | **5.4±0.1*** |
| **16:0 iso** | **1.9±0.2** | **1.6±0.1** | **1.2±0.1** | **1.1±0.6** | **2.7±0.0** | **1.1±0.1*** |
| **17:0 iso** | **3.6±0.2** | **4.5±0.2*** | **5.3±0.3** | **4.9±1.9** | **3.2±0.1** | **4.2±0.1*** |
| **18:0 iso** | **1.3±0.1** | **0.8±0.1** | **0.8±0.1** | **0.6±0.4** | **2.0±0.0** | **0.6±0.0*** |
| **19:0 iso** | **1.5±0.1** | **1.5±0.1** | **2.4±0.1** | **2.1±0.8** | **1.6±0.0** | **1.5±0.0** |
| **Anteiso-BCFA** |  |  |  |  |  |  |
| **15:0 anteiso** | **40.5±0.6** | **40.7±0.4** | **38.0±1.1** | **38.1±0.0** | **37.7±0.2** | **42.2±0.3*** |
| **17:0 anteiso** | **14.1±0.2** | **14.1±0.1** | **13.7±0.8** | **13.6±0.0** | **11.9±0.1** | **16.9±0.5*** |
| **19:0 anteiso** | **4.0±0.2** | **2.9±0.2*** | **3.9±0.2** | **3.5±0.0** | **3.7±0.0** | **3.8±0.0** |
| **UFAs** |  |  |  |  |  |  |
| **18:2ω6, 9c** | **1.0±0.1** | **1.3±0.0** | **1.2±0.1** | **1.4±0.1** | **0.0±0.0** | **1.1±0.1** |
| **18:1ω9c** | **4.1±0.5** | **4.3±0.6** | **3.7±0.7** | **3.9±0.0** | **4.1±0.2** | **3.2±0.2*** |
| **18:1ω7c** | **1.2±0.1** | **1.4±0.1** | **1.5±0.4** | **1.5±0.0** | **1.4±0.0** | **1.3±0.1** |
| **20:1ω9c** | **2.4±0.1** | **2.2±0.1** | **2.6±0.2** | **2.5±0.0** | **2.5±0.1** | **2.0±0.2** |
| **SFAs** |  |  |  |  |  |  |
| **16:0** | **2.5±0.1** | **2.7±0.2** | **2.4±0.4** | **2.7±0.0** | **2.4±0.0** | **2.4±1.0** |
| **18:0** | **8.6±0.1** | **8.6±0.2** | **8.9±0.8** | **9.4±0.0** | **11.5±0.3** | **8.8±3.9*** |
| **20:0** | **4.0±0.3** | **3.2±0.4** | **3.8±0.0** | **4.0±0.0** | **5.9±0.0** | **3.0±1.8*** |

****P* = 0.03 vs. parent.**
